# Supplementary material for: Next-Generation Sequencing-Based Monitoring of Intestinal Bacteria and Bacteriophages Following Fecal Microbiota Transplantation in Inflammatory Bowel Diseases
Source: Pathogens. 2023 Dec 11;12(12):1438. doi: 10.3390/pathogens12121438 (PMC10745900; doi:10.3390/pathogens12121438)
Supplement: Supplementary file 1 [file pathogens-12-01438-s001.zip › pathogens-2730011-supplementary.pdf]

Table S1. List of primers for intestinal dsDNA viruses in the studied panel NGS panel.

Name of virus	Strain	Primer names	Primer sequences (5'-3')	Length of PCR product	Reference
**Bacteriophages**					
E.coli X52686.1 Bacteriophage T4 rIIA gene and ORF rIIA.1	T4	T4F	CCATCCATAGAGAAAA-TATCAGAACGA	100	Ninove et al., 2011
		T4R	TAAA-TAATTCCTCTTTTCCCAGCG		
E.coli	MS2	MS2F	CTCTGAGAGCGGCTCTATTGGT	100	Ninove et al., 2011
		MS2R	GTTCCCTACAAC-GAGCCTAAATTC		
Klebsiella pneumonia phage	kpv71 KU666550 (Autographviridae)	71F_NcoI	AGTGCCCATGGCATTAATT-AGATTAGTAGCTC		Solovieva et al., 2018; Knecht et al, 2019
		71R_XhoI	CATACTCGAGATACAC-CTCCCAGGCTACCTG		
	Kpv74	74F_NcoI	GAATCCATGGCACTAGTAGAT-TTAGTGAGG		Solovieva et al., 2018 A.Latka, 2019
		74R_XhoI	ATTACTCGAGCACATT-GCACAGCAAGTACG		
Salmonella enteric phages	SEN22	F4c5-F	CAACTTGCGACTGCTCTTTG	474	Mikalová et al.,
		F4c5-R	CGAAGGAAGCGTTAGACCTG		2017
	SEN2	F3c7-F	GTTGGGTGGAAGAAGCTGAA	413	Mikalová et al.,
		F3c7-R	AGCATCTGGCCCTGTATCTG		2017
	SEN8	F20c3-F	TTCAGTGGTTGGTTCCATGA	471	Mikalová et al.,
		F20c3-R	GCGTTAAGAAGCCAGAAACG		2017
	SEN8	F20c5-F	TTATTTGCTGCGCTGACATC	494	Mikalová et al.,
		F20c5-R	TCAGAAGAAGGCCTGGCTAA		201
	SEN23	F6c1-F	GGCCGATCAGTTGCTTAAAA	232	Mikalová et al.,
		F6c1-R	CTCATGCCCCAACATTTTCT		2017
	SEN4	F9c2-F	GCCGTCTGATAACCCAGAAA	456	Mikalová et al.,
		F9c2-R	CGTTTTCCAGATCAGCCTGT		2017
	SEN34	F38c2-F	AAGTGGGGAACTGCTGAAGA	487	Mikalová et al.,
		F38c2-R	AACGCTGCCATAAGCTGACT		2017
	SEN1	F1c3-F	AAAGGCCGGTTAGGTAGCTC	456	Mikalová et al.,
		F1c3-R	ATCGCTCGCATGTTTAACG		2017
	SEN22	F4c4-F	AATGGATGCAGTCAGGGAAG	421	Mikalová et al.,
		F4c4-R	GTCAATCATCGCGTTTTCCT		2017
	SEN5	F10c1-F	TTCGCAAATGAAATCGAGTG	443	Mikalová et al.,
		F10c1-R	ATCGGCAACTTACCGTCATC		2017
	SEN23	F6c4-F	GTTGTTCAGGCCGTTGATTT	284	Mikalová et al.,
		F6c4-R	GTACATCGCCTGAAGGGAGA		2017
	SEN34	F38c1-F	GTTTATGGCGCTGAAAAGGA	457	Mikalová et al.,
		F38c1-R	GTTGTTCAGGCCGTTGATTT		2017
Bacteroides	CrAssphage	CrassPhF	AGACGCGATGAAGAACTGCT	590	Yangpeng Li et al., 2021
		CrassPhR	CCATCGGGAGCAGTAAGACC	500	
		CrPhPolF	CGGCGGGTTAATCAAAA-TAGAA	500	Yangpeng Li et al., 2021
		CrPhPolR	GCGGAGAACCCCATTTATTAA-TAAG		

**Eukaryotic cell viruses**					
Cytomegalovirus (DNA)	AD169, Toledo, Towne	UL55N_F	GGA TCT GGT GCC TGG TAG TC	250	
		UL55N_R	CGA ATA AGA TCC GTA CCC TG		
	AD169, Toledo, Towne	UL55C_F	TGT TCT GGC AAG GTA TCA AGC A	250	
		UL55C_R	GTG AAC TGC AGC TGG GCG TA		
	AD169	UL73_F	TTC GGT CGG TCA ACA TCG TAA G	361	Grosjean et al., 2009
		UL73_R	GGT GGT TGC AGT AAA GTT CTG GA		
	Toledo	UL75_F	TCC TGG GAT CCT TTC TCT CCT TCT	429	
		UL75_R	ATG GGT CTC CCG TAG GTG TTG		
Varicella Zoster (DNA) Human herpesvirus 3 (VZV, strain Dumas) complete genome - X04370<br>Human herpesvirus 3 DNA, complete genome, strain: Oka, sub_strain: pOka - AB097933.1	Dumas, Oka	vzv49f	TGAGCAACTTGATCCGTGTC	347	
		vzv49r	CCTCGCCATAAAGCCACTAC		
		vzv62f	ACAAACACAGGGGTTGTTCG	339	
		vzv62r	GCGTTTTATTACTGTCGACC		
		vzv52f	CGGTGGACACACAGAAAGAG	360	Jin et al., 2017
		vzv52r	GCCCTGAACCAGTTCTACCA		
		vzv11f	ACCCACCATCATTTGAGTCC	350	
		vzv11r	GCCGTTTGAGACCGATGATA		
Adenovirus		AdV ACF F1	GGT CTG GTG CAA TTC GCC	135	
		AdV ACF R1	CAC GGG CAC AAA ACG CA		
		AdV ACF F2	ACC TGG GCC AAA ACC TTC TC	75	
		AdV ACF R2	CGT CCA TGG GAT CCA CCT C		Ebner et al, 2005
		AdV ACF F3	CCC GTG TTT GAC AAC GAA GG	112	
		AdV ACF R3	TTA GAG CTA GGC ATA AAT TCT ACA GCA		
		AdV BDE_F	ACA TGC ACA TCG CCG G	143	
		AdV BDE_R	CGG TCS GTG GTC ACA TC		
Adenovirus	Hexon gene	ADHEX2F1	AACACCTACGAC-TACATGAATGG	133-155	Ylihärsilä et al., 2013<br>Ebner K, et al., 2005
		ADHEX2F2	AAAAACACCTACGAC-TACATGAACGG	130-155	
		ADHEX2F3	AACACCTACGATTA-TATGAACAAGC	133-157	
		Adhex1R	ACATCCTTCCTGAAGTTCCA	385-404	

Sources:

Ninove L, Nougairede A, Gazin C, Thirion L, Delogu I, Zandotti C, Charrel RN, De Lamballerie X. RNA and DNA bacteriophages as molecular diagnosis controls in clinical virology: a comprehensive study of more than 45,000 routine PCR tests. PLoS One. 2011;6(2):e16142. doi: 10.1371/journal.pone.0016142.

Solovieva EV, Myakinina VP, Kislichkina AA, Krasilnikova VM, Verevkin VV, Mochalov VV, Lev AI, Fursova NK, Volozhantsev NV. Comparative genome analysis of novel Podoviruses lytic for hypermucoviscous Klebsiella pneumoniae of K1, K2, and K57 capsular types. Virus Res. 2018;243:10-18. doi: 10.1016/j.virusres.2017.09.026.

Latka A, Lemire S, Grimon D, Dams D, Maciejewska B, Lu T, Drulis-Kawa Z, Briers Y. Engineering the Modular Receptor-Binding Proteins of *Klebsiella* Phages Switches Their Capsule Serotype Specificity. mBio. 2021 May 4;12(3):e00455-21. doi: 10.1128/mBio.00455-21.

Mikalová L, Bosák J, Hříbková H, Dědičová D, Benada O, Šmarda J, Šmajs D. Novel Temperate Phages of Salmonella enterica subsp. salamae and subsp. diarizonae and Their Activity against Pathogenic S. enterica subsp. enterica Isolates. PLoS One. 2017;12(1):e0170734. doi: 10.1371/journal.pone.0170734.

Li Y, Gordon E, Shean RC, Idle A, Deng X, Greninger AL, Delwart E. CrAssphage and its bacterial host in cat feces. Sci Rep. 2021;11(1):815. doi: 10.1038/s41598-020-80076-9.

Grosjean J, Hantz S, Cotin S, Baclet MC, Mengelle C, Trapes L, Virey B, Undreiner F, Brosset P, Pasquier C, Denis F, Alain S. Direct genotyping of cytomegalovirus envelope glycoproteins from toddler's saliva samples. J Clin Virol. 2009; 46 Suppl 4:S43-8. doi: 10.1016/j.jcv.2009.08.018.

Jin L, Xu S, Maple PAC, Xu W, Brown KE. Differentiation between wild-type and vaccines strains of varicella zoster virus (VZV) based on four single nucleotide polymorphisms. Epidemiol Infect. 2017; 145(12):2618-2625. doi: 10.1017/S0950268817001509.

Ebner K, Suda M, Watzinger F, Lion T. Molecular detection and quantitative analysis of the entire spectrum of human adenoviruses by a two-reaction real-time PCR assay. J Clin Microbiol. 2005; 43(7):3049-53. doi: 10.1128/JCM.43.7.3049-3053.2005.

Ylihärsilä M, Harju E, Arppe R, Hattara L, Hölsä J, Saviranta P, Soukka T, Waris M. Genotyping of clinically relevant human adenoviruses by array-in-well hybridization assay. Clin Microbiol Infection. 2013; 19(6): 551-557. doi: 10.1111/j.1469-0691.2012.03926.x
